# Supplementary material for: SARS-CoV-2 Spike Protein 1 Causes Aggregation of α-Synuclein via Microglia-Induced Inflammation and Production of Mitochondrial ROS: Potential Therapeutic Applications of Metformin
Source: Biomedicines. 2024 May 31;12(6):1223. doi: 10.3390/biomedicines12061223 (PMC11200543; doi:10.3390/biomedicines12061223)
Supplement: Supplementary file 1 [file biomedicines-12-01223-s001.zip › Supplementary Table S1.pdf]

# Title: SARS-CoV-2 spike protein 1 causes aggregation of α-synuclein via microglia-induced inflammation and production of mitochondrial ROS *in vitro* and *in vivo*: potential therapeutic applications of metformin

Moon Han Chang*, Jung Hyun Park*, Hae Kyung Lee, Ji Young Choi & Young Ho Koh

Division of Brain Diseases Research, Department of Chronic Disease Convergence Research. Korea National Institute of Health, 187 Osongsaengmyeong2(i)-ro, Osong-eup, Heungdeok-gu, Cheongju-si, Chungcheongbuk-do 28159, Republic of Korea

*These authors equally contributed to this work

Correspondence should be addressed to Young Ho Koh, Division of Brain Diseases Research, Korea National Institute of Health, Tel.: +82 43 719 8630; Fax: +82 43 719 8602; Email; koyoungho122@gmail.com

**Supplementary Table S1. Lists of antibodies**

| Antibody | Manufacturer | Catalog |
|---|---|---|
| α-synuclein (5G4) | MERCK | MABN389 |
| P-α-synuclein (Ser129) | CST | 23706S |
| α-synuclein (monomer) | Abcam | ab212184 |
| TNF- α | CST | 11948S |
| IL-1β | CST | 12242S |
| Iba-1 | MilliporeSigma | SAB2702364 |
| CD163 | Abcam | ab182422 |
| β-actin | Sigma | A5316 |
| His-tag | R&D systems | MAB050 |
