# Supplementary figures and images for: SARS-CoV-2 Spike Protein 1 Causes Aggregation of α-Synuclein via Microglia-Induced Inflammation and Production of Mitochondrial ROS: Potential Therapeutic Applications of Metformin
Source: Biomedicines. 2024 May 31;12(6):1223. doi: 10.3390/biomedicines12061223 (PMC11200543; doi:10.3390/biomedicines12061223)

Supplementary figure. 1

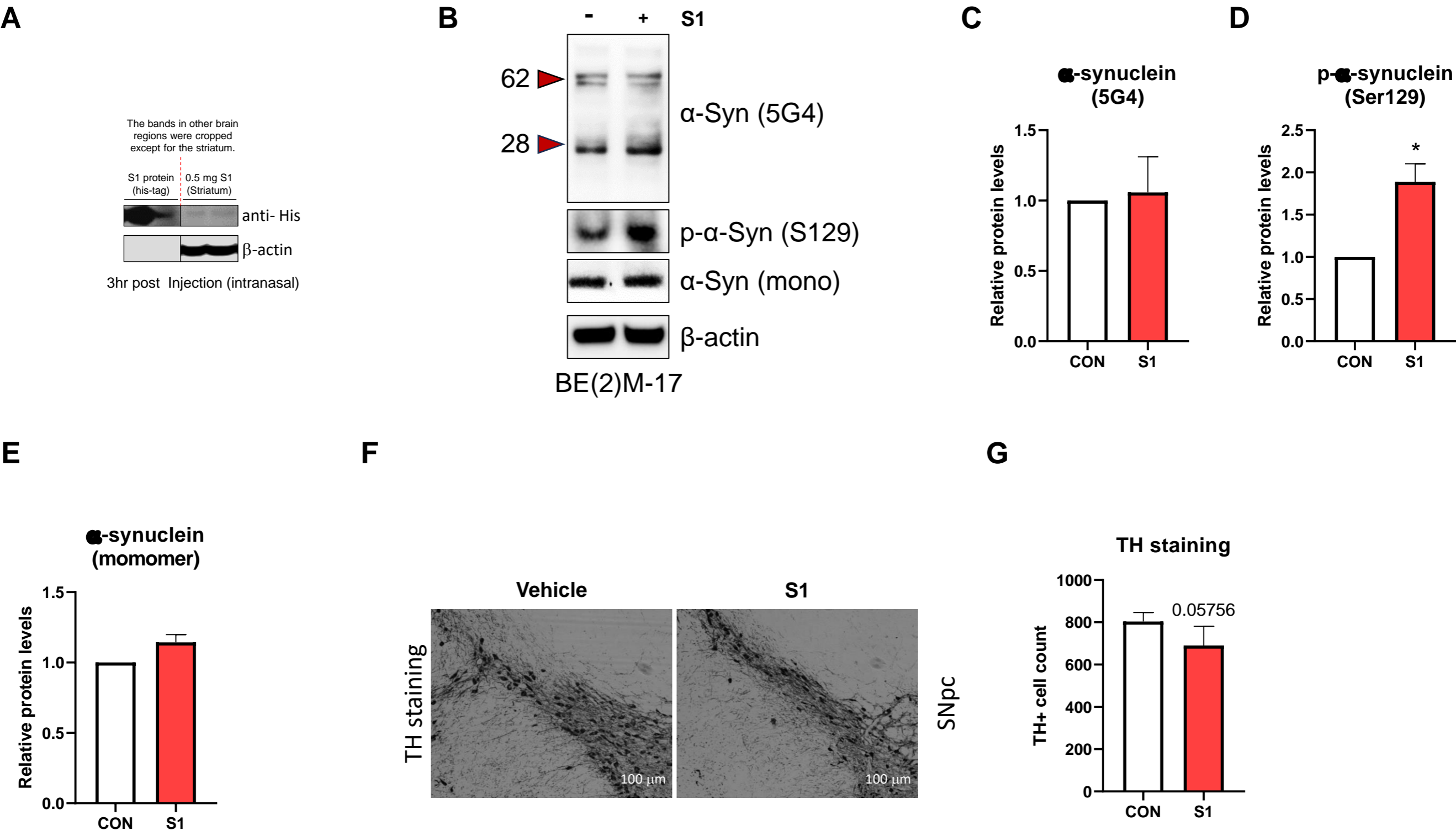

Supplement: Supplementary file 1 [file biomedicines-12-01223-s001.zip › Figure S1.pdf]
